# Intra-Cranial Abscess and Middle Ear Disease

**Published:** 1903-10-24

**Authors:** 


### INTRA-CRANIAL ABSCESS AND MIDDLE-EAR DISEASE.

The difficulties in the way of localising a cerebral abscess due to middle-ear disease, even when only one ear is affected, are well known. Where both ears are affected the diagnosis is made more difficult still. A case reported by Dr. E. Gruening[1] illustrates what may happen in such circumstances. The patient was a girl ten years old, who first came under notice with headache, drowsiness, and vomiting. There was a history of sudden onset of acute pain in the left side of the head, which came on ten days previously. Five days later there were chills and fever, severe headache, and purulent discharge from the left ear. On examination there was found to be perforation of the left drum membrane, with tenderness, redness and œdema over the left mastoid process. The right membrana tympani was red and swollen. An operation was performed. The left mastoid antrum was opened and found to contain pus and granulations. Thorough curettage was carried out, and the wound was packed with gauze. The patient was much improved, but on the following day discharge was noticed coming from the right ear; this continued for a week and then ceased. Nine days after symptoms of cerebral abscess developed, and the left temporo-sphenoidal lobe was explored, but no pus was found. The symptoms increased, and a week later the left temporo-sphenoidal lobe, left cerebellum and lateral sinus were explored, with a negative result. The symptoms grew more pronounced, with slow pulse and respirations, double optic neuritis, vomiting and so forth, and the patient died. At the post-mortem examination an abscess was found in the right lobe of the cerebellum. A very misleading circumstance in this case was that the perforation in the right membrana tympani had healed.

[1] Medical Record, October 3.

